# Carotidynia presenting as an atypical cause of unilateral neck pain in the emergency department

**DOI:** 10.1093/omcr/omad094

**Published:** 2023-09-25

**Authors:** Muhammad Akbar Baig, Sadia Asad

**Affiliations:** Department of Emergency Medicine, Aga Khan University Hospital, Karachi, Pakistan; Department of Emergency Medicine, Aga Khan University Hospital, Karachi, Pakistan

## Abstract

Carotidynia is a rare presentation of atypical neck and face pain, which is due to inflammation around the carotid artery. Symptoms can get aggravated by head and neck movements, jaw movements and deglutition. It is usually a self- limiting illness, and it is treated conservatively with analgesics. Because of it is rarity, and partly due to physician’s lack of understanding, it remains underdiagnosed. Our case report aims to shed light on the importance of how its diagnosis cannot be missed.

## INTRODUCTION

Carotidynia, also known as carotid artery pain syndrome or Fay syndrome, is a rare condition characterized by unilateral or bilateral pain in the neck, face and head caused by inflammation of the carotid artery. The condition typically affects individuals in their forties or fifties, and it is more common in women than men. The etiology of carotidynia remains unclear, but it is believed to be caused by infectious or inflammatory processes affecting the vascular wall [1, 2].

## CASE REPORT

**Figure 1:**
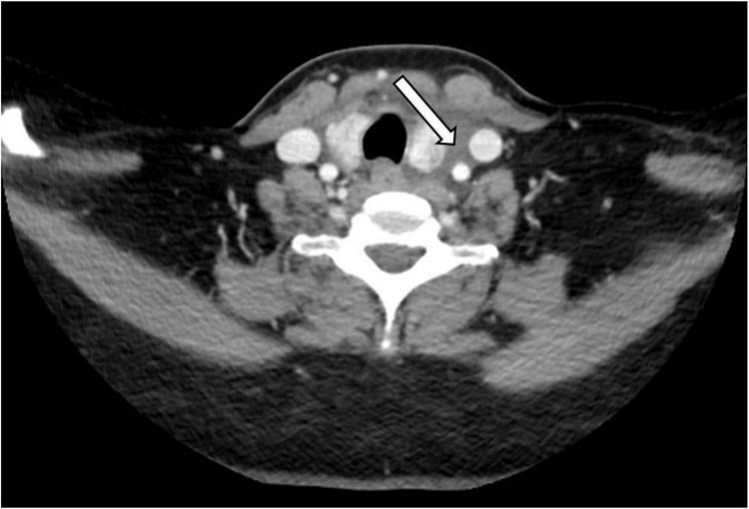
Axial section of CT-Neck showing eccentric, anteromedial inflammation surrounding the carotid arteries (white arrow).

**Figure 2:**
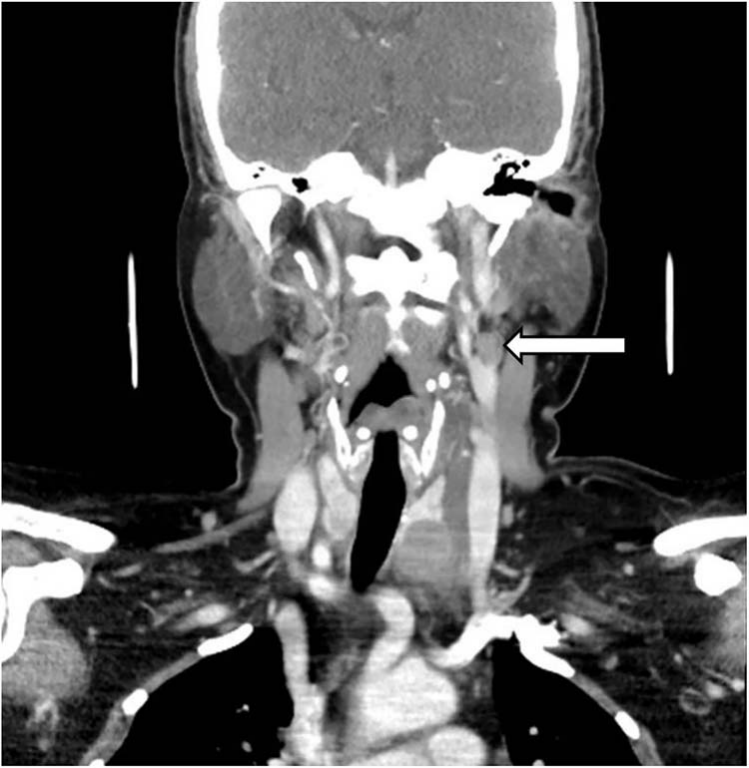
Coronal section of CT neck, showing a anteromedial inflammation around the carotid bifurcation (white arrow).

A 49-year-old with hypertension and Graves disease presented to ED with complaints of left sided neck discomfort for the last 6 days. She later began developing fever and progressively increasing left-sided neck swelling with episodic pain that began radiating to the left half of her face. She also complained of dysphagia. The patient had no history of trauma, recent infection or vascular disease. She denied any visual changes, difficulty speaking or weakness in her limbs. She was compliant with her lisinopril and thyroxine. Her vitals showed a heart rate of 95 beats/min, blood pressure of 157/85 mmHg and respiratory rate of 18/min with normal room air oxygen saturations. Physical examination revealed tenderness over the entire extent of left carotid artery, but no palpable mass or bruit was noted. Neurological examination was unremarkable. She was treated with analgesics. After engaging in a shared decision-making process with the patient’s family and physicians, it was collectively agreed that, due to the presence of severe pain directly overlying the carotid artery, a comprehensive examination using an enhanced computerized tomography scan of her neck would be conducted. The computed tomography (CT) scan showed eccentric, anteromedial thickening around and below the carotid bifurcation, extending inferiorly along the fascial planes between left thyroid lobe and carotid vasculature into the superior mediastinum (Figs 1 and 2). Overall appearances were suggestive of carotidynia. Patient was admitted for pain management, and her symptoms gradually improved. She was discharged on non-steroidal anti-inflammatory drugs (NSAIDS) with advice to follow-up with a vascular specialist.

## DISCUSSION

The pathophysiology of carotidynia remains unclear. Several hypotheses have been proposed, including infectious, autoimmune and traumatic etiologies. The most widely accepted theory is that carotidynia is an inflammatory condition affecting the vascular wall of the carotid artery. The diagnosis of carotidynia is based on clinical presentation and imaging findings. Patients typically present with unilateral or bilateral pain in the neck, face and head, which may be aggravated by swallowing or turning the head. Physical examination may reveal tenderness over the affected carotid artery, but no palpable mass or bruit is typically noted. Carotid Doppler ultrasound (US) may show increased blood flow velocity and wall thickening of the affected carotid artery [3].

Lecler *et al*. have debated over the term carotidynia that it should be replaced by transient perivascular inflammation of the carotid artery (TIPIC syndrome) due to the consistent radiographic finding of inflammation surrounding the carotid trunk at or near the bifurcation. To diagnose the TIPIC syndrome, the following criteria needs to be fulfilled, which include: having sharp pain over the carotid arteries with or without radiation to head, peculiar perivascular infiltration, exclusion of other vascular and non-vascular causes by radiological imaging and spontaneous or anti-inflammatory induced recovery within 14 days [4]. However, there continues to remain controversy between carotidynia versus TIPIC syndrome, and these two terms are still used synonymously by many clinicians when investigating differentials for idiopathic unilateral neck pain [5].

The choice of imaging modality depends on various factors such as the severity of symptoms, clinical presentation and availability of resources. Duplex Doppler US is a commonly used initial imaging modality for evaluating carotidynia. It can assess blood flow and detect any structural abnormalities in the carotid artery, such as plaque buildup or narrowing. Enhanced CT scan can visualize stenosis, aneurysm or dissection in the carotid artery. It is particularly useful in emergency situations when a quick assessment is required. Other advance imaging techniques include magnetic resonance imaging, which can provide further detailed images of the carotid artery and surrounding structures in the neck and positron emission tomography, which evaluates the metabolic activity of the carotid artery and helps differentiate between active inflammation and non-inflammatory causes of carotid pain [3, 6].

The treatment for carotidynia is based on symptom relief and addressing any underlying conditions. NSAIDs are typically the first-line therapy for pain relief, and corticosteroids may be considered in refractory cases [7]. Infection or other inflammatory conditions should be ruled out and treated if present. Prognosis for carotidynia is generally good, with most patients experiencing resolution of symptoms within several weeks to months. Recurrence is uncommon but may occur in some patients [6].
